# Optimised neural network model for river-nitrogen prediction utilizing a new training approach

**DOI:** 10.1371/journal.pone.0239509

**Published:** 2020-09-28

**Authors:** Pavitra Kumar, Sai Hin Lai, Nuruol Syuhadaa Mohd, Md Rowshon Kamal, Haitham Abdulmohsin Afan, Ali Najah Ahmed, Mohsen Sherif, Ahmed Sefelnasr, Ahmed El-shafie

**Affiliations:** 1 Department of Civil Engineering, Faculty of Engineering, University of Malaya, Kuala Lumpur, Malaysia; 2 Department of Biological and Agricultural Engineering, Faculty of Engineering, Universiti Putra Malaysia, Selangor, Malaysia; 3 Institute of Research and Development, Duy Tan University, Da Nang, Vietnam; 4 Institute for Energy Infrastructure (IEI), Universiti Tenaga Nasional (UNITEN), Kajang, Selangor, Malaysia; 5 National Water and Energy Center, United Arab Emirates University, Abu Dhabi, United Arab Emirates; 6 Civil and Environmental Eng. Dept., College of Engineering, United Arab Emirates University, Abu Dhabi, United Arab Emirates; Chinese Academy of Sciences, CHINA

## Abstract

In the past few decades, there has been a rapid growth in the concentration of nitrogenous compounds such as nitrate-nitrogen and ammonia-nitrogen in rivers, primarily due to increasing agricultural and industrial activities. These nitrogenous compounds are mainly responsible for eutrophication when present in river water, and for ‘blue baby syndrome’ when present in drinking water. High concentrations of these compounds in rivers may eventually lead to the closure of treatment plants. This study presents a training and a selection approach to develop an optimum artificial neural network model for predicting monthly average nitrate-N and monthly average ammonia-N. Several studies have predicted these compounds, but most of the proposed procedures do not involve testing various model architectures in order to achieve the optimum predicting model. Additionally, none of the models have been trained for hydrological conditions such as the case of Malaysia. This study presents models trained on the hydrological data from 1981 to 2017 for the Langat River in Selangor, Malaysia. The model architectures used for training are General Regression Neural Network (GRNN), Multilayer Neural Network and Radial Basis Function Neural Network (RBFNN). These models were trained for various combinations of internal parameters, input variables and model architectures. Post-training, the optimum performing model was selected based on the regression and error values and plot of predicted versus observed values. Optimum models provide promising results with a minimum overall regression value of 0.92.

## Introduction

Human activities have altered the presence of nitrogenous compounds in rivers. Industrialization and the intense use of fertilizers in agricultural fields represent the main causes of the enhancement of these compounds in rivers’ water. The excessive use of high nitrogen content fertilizers has increased the rate of release of these compounds, especially nitrate-nitrogen, in the environment. As such, adverse impacts on the environmental system and human health have been observed [1, 2]. In rivers, surplus nitrogenous compounds lead to magnification of algae on the water surface [3], which restricts the contact of water with light and air and also reduces the oxygen supply for aquatic lives. These compounds lead to different types of cancer [4] and two types of birth defects [5, 6]. Nitrates in drinking water causes “blue baby syndrome” in infants [4] and also various tumours in the human body [4, 7]. Proper monitoring and maintenance of the water quality is required to control the nitrogen level in rivers. Lack of monitoring systems may result in an abrupt rise of nitrogen concentrations in rivers that could lead to the closure of water treatment plants as most of the plants are not designed for the complete removal of nitrogen. In Malaysia, an abrupt rise in nitrogenous compounds levels in various rivers has led to the frequent closure of water treatment plants [8]. These plants often have complicated processes and require total control over the system [9, 10]. Information on the concentrations of such pollutants are therefore, critical to ensure the continuity of operations of these treatment plants. Hence, there comes a need for a model, which predicts the level of nitrogenous compounds in advance. In the last few years, a number of models have been designed to predict hourly, daily and monthly data for different pollutants other than nitrogen in Malaysian rivers.

Artificial Neural Network (ANN) models, a computational intelligence model, have been extensively used for prediction over the last few decades [11]. These models form a network similar to the neurons system in the human brain. They mathematically relate the input to the desired output, forming a completely data-driven model. An ANN model trains itself with the historical data of the desired output and using the training parameters, it predicts the upcoming data. It has various internal parameters (such as hidden layers, nodes in hidden layers, maximum epochs, spread values, etc.) that need to be adjusted to get the results with high accuracy. ANN has the unique feature of learning the crests and troughs of the historical data used for a model training. He, Oki [12] reported that, ANN models are used for reservoir operations [13–17], water resources management [18, 19] and hydrological processes [20, 21].

Several studies, including [15, 22–25], used ANN for predicting nitrogenous compounds in rivers across the world. As used by Fiyadh, AlSaadi [26], authors have searched on Science Direct and Google Scholar to find these relevant studies. Most of these studies have not considered the application of different architectures of ANN, such as multilayer, RBFNN and GRNN. In addition, none of the models have been trained for the Malaysian hydrological conditions. An ANN model trained for a particular set of input data for some locations cannot be used efficiently at different locations as the pattern of the historical input data may not be same as the previous ones. In other words, such ANN models are site specific and may not be implemented before further training on other sites. Hence, there is a need for the development of an efficient model for the Malaysian rivers.

In Malaysia, ANN models have been used to predict various hydrological parameters, but none have addressed the prediction of the nitrogenous compounds in Malaysian rivers. Unlike available literature, this study proposes a new training approach and a selection procedure of the optimum performing ANN model. The developed model fulfils the existing needs for nitrate-N and ammonia-N predictions in Malaysian rivers.

The objectives of this investigation are to present the application of ANN for the prediction of the monthly average nitrate-N and monthly average ammonia-N levels in the Langat River basin in Selangor, Malaysia.

## Artificial neural network

**Fig 1:**
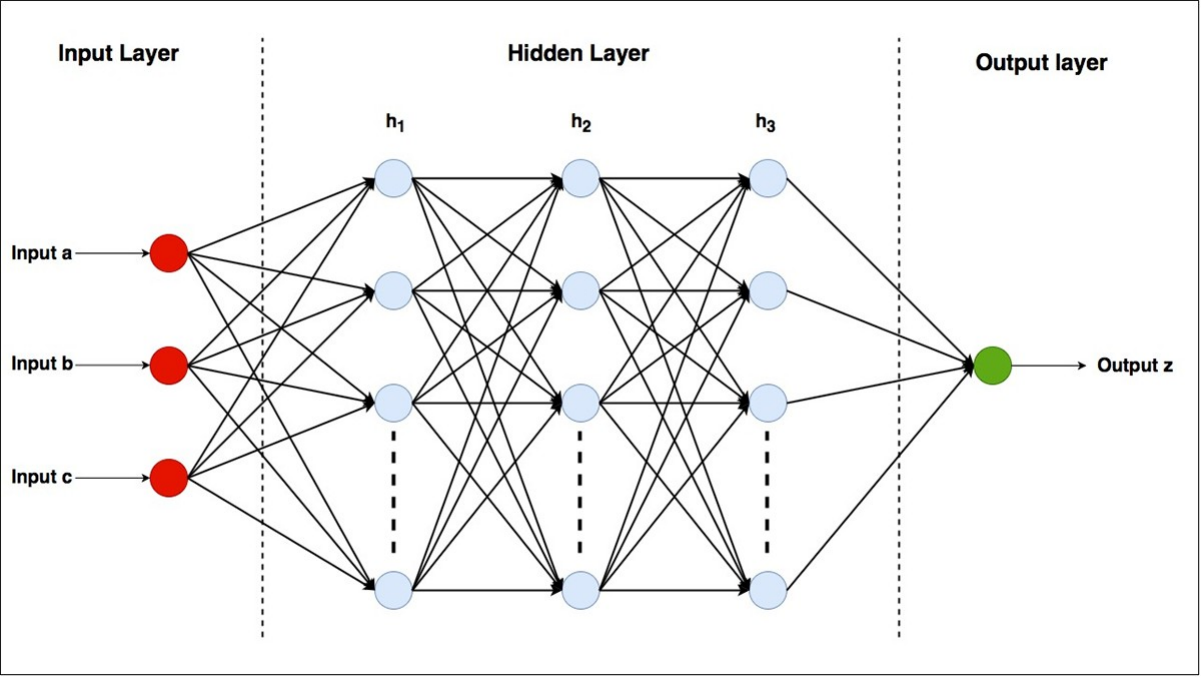
Basic structure of ANN model.

ANN is black-box model which establishes a relation between input variables and desired output variables [27]. Inside the black-box, a network is formed within the neurons which is similar to that of the nervous system in human brain [23, 24, 28]. The advantages of the ANN models include: (i) generalization of the unseen situations [29, 30], (ii) ability to perform model-free function estimations, (iii) ability to learn from data relationships that are not otherwise known and, (iv) ability of handling non-linear functions [31, 32]. The ANN model consists of input layer, hidden layer and output layer [33]. Input variables are provided in the input layer; which are then passed to the inner hidden layers [34], where the weights corresponding to each input variables are adjusted to get a better relationship with the desired output. Fig 1 represents the basic structure of ANN models. In this model there are three input variables, a, b, and c; with three hidden layers, h_1_, h_2_ and h_3_; and one output layer z. In the current study, a, b, c, and z represents the rainfall, water level, discharge, and nitrate-N or ammonia-N, respectively. General Regression Neural Network (GRNN), multilayer perceptron and Radial Basis Function Neural Network (RBFNN) composed the three model architectures applied in the current study. These three ANN architectures are the examples of feed-forward ANNs [35]. Training and testing of these models were conducted on Matlab platform.

Based on non-parametric regression, GRNN is considered as an improved technique in ANN. It has the same number of the neurons in the input layer as the number of input variables, and the same number of neurons in the output layer as the number of output parameters. GRNN uses supervised training; which allows the model to compare the predicted output with the observed output, provided at the time of training [36, 37]. Multilayer perceptron is the most popular [38, 39] and efficient ANN architecture used nowadays in the field of modelling [31, 35]. It follows supervised training and is mostly used for modelling complex relationship between different stochastic variables [31]. Multilayer perceptron has the number of neurons in input and output layers, as defined by the user during training. RBFNN is mostly used for the remotely sensed data as it has been proved to be good function approximators and classifiers. RBFNN is considered as an alternative of the other ANN architectures, as it reduces the training time. The number of neurons in RBFNN depends on the number of training patterns [40].

## Study area

This study is based on the Langat River basin in Selangor, Malaysia. This basin has been selected as the Langat River has been facing the problem of high nitrogen content between 2012 to 2015, which led to the frequent suspension of different water treatment plants during that time period. As stated by Selangor Water Management Authority, Malaysia, the level of ammonia-N in the Langat River has exceeded 7.0 mg/l several times between 2012 and 2015 [41], resulting in the suspension of treatment plant operations. A study by AYERS, PENG [42], stated that the atmospheric deposition of oxides of sulphur and nitrogen in Petaling Jaya, a city near the Langat River basin, lies within the range 277–480 meq-m^-2^yr^-1^, with nitrogen species contribution of 56%.

This basin has a catchment area of about 2400 km^2^. The Langat River supplies about 65% of the total water usage in the Selangor state. The Langat Dam (area 41.0 km^2^) and the Semenyih Dam (area 56.6 km^2^) are the two major reservoirs supplying water to the state [43]. As per the 2013 analysis, the Langat River basin has a forest area of about 48,285.0 ha, an agricultural area of about 142,387.916 ha and a developed area of about 69,056.1 ha [44]. About 72% of the soil in Malaysia is acidic and highly weathered (Ultisols and Oxisols) [45], which requires fertilizers for agriculture. The main fertilizers used in Malaysia are urea, ammonium sulphate, calcium ammonium nitrate, phosphate rock, super phosphates, ammonium phosphate, potassium chloride, potassium sulphate and NPK, NP and PK compound fertilizers [45]. Along with the agricultural runoff, livestock wastes also increases the nitrogen content in rivers. Livestock production in Malaysia consists of pork, poultry meat and eggs; and it has to import milk, beef and mutton.

The Langat River basin has a hot and humid tropical climate with a 27°C average annual temperature, which is uniform throughout the year and a 2470 mm average annual rainfall distributed throughout the year [46].

**Fig 2:**
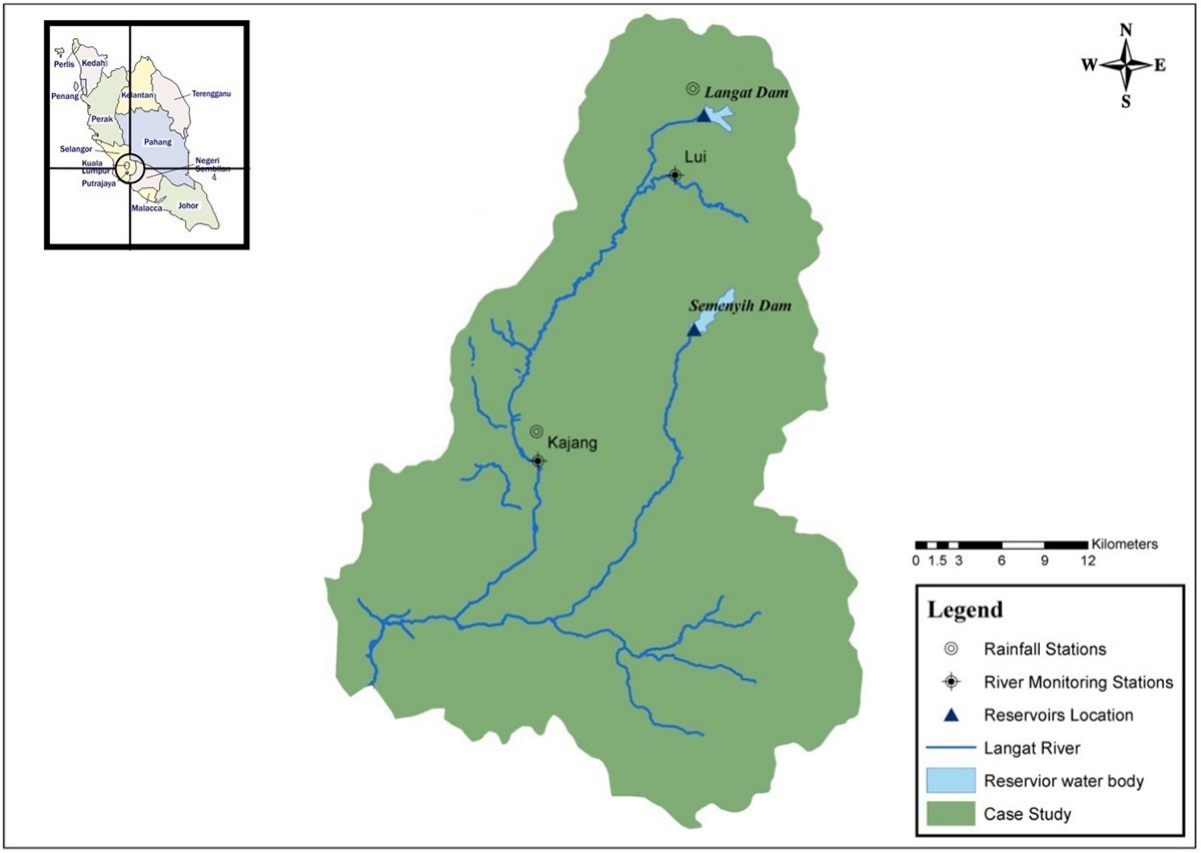
Langat River basin [47]. Reprinted from [47] under a CC BY license, with permission from PLOS ONE, original copyright 2017.

Within the course of the Langat River flow, data from two water quality stations (Lui and Kajang) were acquired from the Department of Irrigation and Drainage, Kuala Lumpur, Malaysia. The water quality station, Lui, is situated at the river Lui, in the upstream region of the Langat River basin, as shown in Fig 2. This region is mainly mountainous and is less populated and hence, has less agriculture and industries activities. The water quality station, Kajang, is situated at the Langat River in Kajang town. This town is densely populated and is located near the capital city, Kuala Lumpur. Within the path of flow from Lui to Kajang, the Langat River receives inflow from various agricultural fields of rubber, paddy and coconuts, and from various industries as well. These inflows increase the nitrogen content in the Langat River, which is clearly reflected in the water quality data of Kajang. Nitrate-N at the Lui station has an average value of 1.34 mg/l (Table 1), which increases to an average value of 7.32 mg/l at the Kajang station. In addition, ammonia-N at the Lui station has an average value of 0.11 mg/l, which reaches 1.96 mg/l, at Kajang station.

**Table 1:** Statistical analysis of the data for both stations.

	Lui Station Data					Kajang Station Data				
	RF (mm)	WL (m)	Q (m^3^/s)	Nitrate-N (mg/l)	Ammonia-N (mg/l)	RF (mm)	WL (m)	Q (m^3^/s)	Nitrate-N (mg/l)	Ammonia-N (mg/l)
**Average**	6.85	76.17	2.19	1.34	0.11	6.89	22.7	12.53	7.32	1.96
**Standard Deviation**	3.57	1.05	1.02	0.87	0.14	3.87	0.34	7.47	6.09	1.93
**Skewness**	0.43	-0.69	1.29	1.01	2.41	0.61	0.34	1.21	0.998	1.39
**Maximum**	16.70	77.41	7.66	5.30	0.64	18.75	23.8	40.60	29.5	8.4
**Minimum**	0.10	74.41	0.68	0.01	0.01	0.00	22.0	2.50	0.02	0.05

## Methodology

### Data collection and interpolation

Water quality (mainly comprising of nitrate-N and ammonia-N), water level (WL) and discharge (Q) data of Lui and Kajang water quality stations and rainfall (RF) data of the nearest rainfall gauge stations of Lui and Kajang were collected. These data were obtained from Department of Irrigation and Drainage (DID), Malaysia, for the period of 1981–2017. The target variables (i.e. nitrate-N and ammonia-N) obtained were measured on monthly basis. To align with the target variables, rest of the data were converted from daily data to monthly data, by considering the 30-day average values as an average value for a particular month. The input variables selected for the current study are RF, WL and Q, as the concentrations of nitrate-N and ammonia-N in rivers depend on rainfall, water flow [48] and depth [22]. Nitrate-N concentration reduces when river receives short and intense rainfall water and it may increase if the rainfall is prolonged one, as water leaches through the soil in the latter case, collecting nitrate-N from the soil. Water flow controls the transformation processes of nitrate-N and ammonia-N i.e. nitrification and denitrification [48]. Czernuszenko [22] reported that the concentration of pollutants depend on depth of the river. Concentration of pollutant is lower for rivers with greater depth.

**Fig 3:**
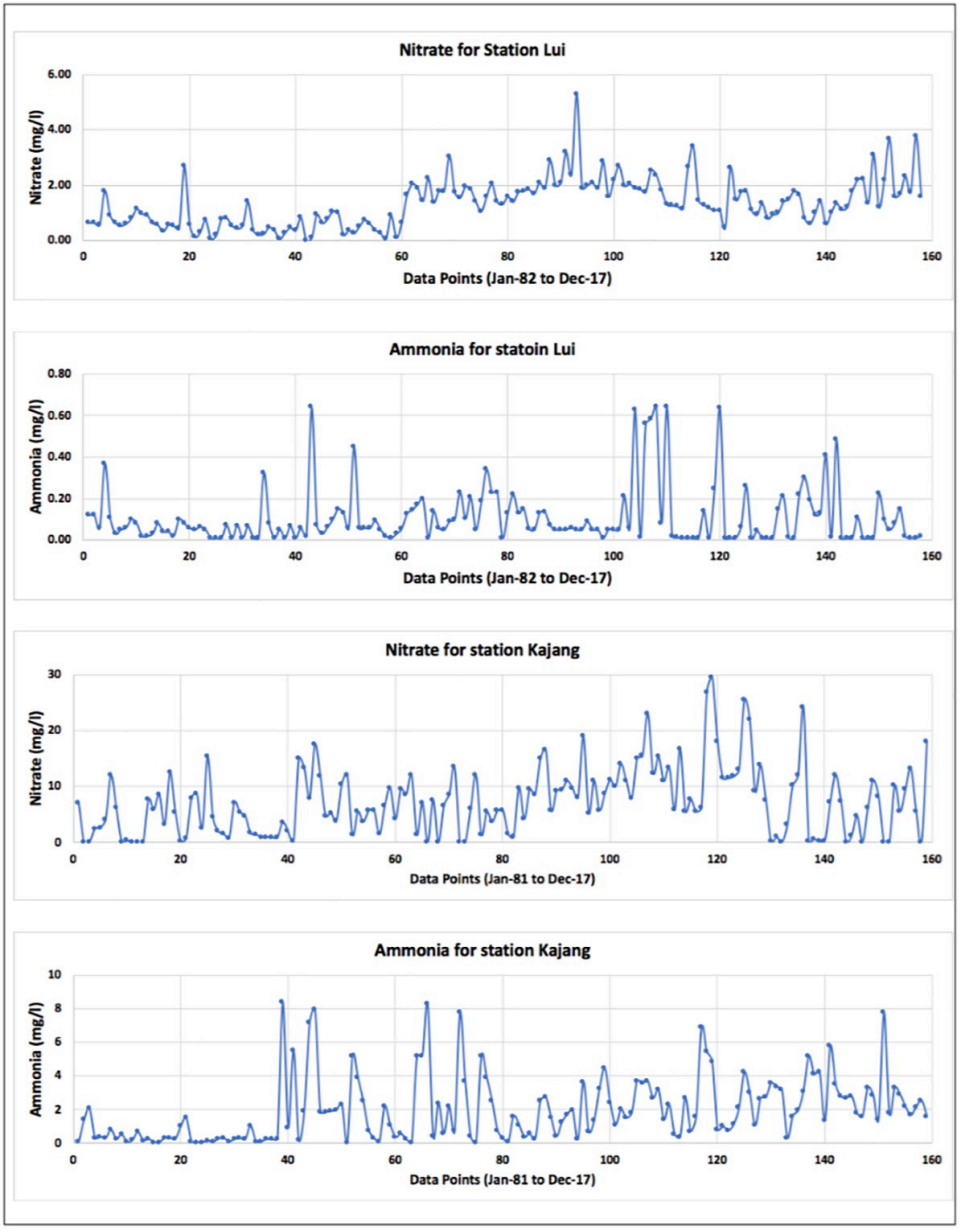
Plot of monthly average interpolated data of nitrate-N and ammonia-N.

**Fig 4:**
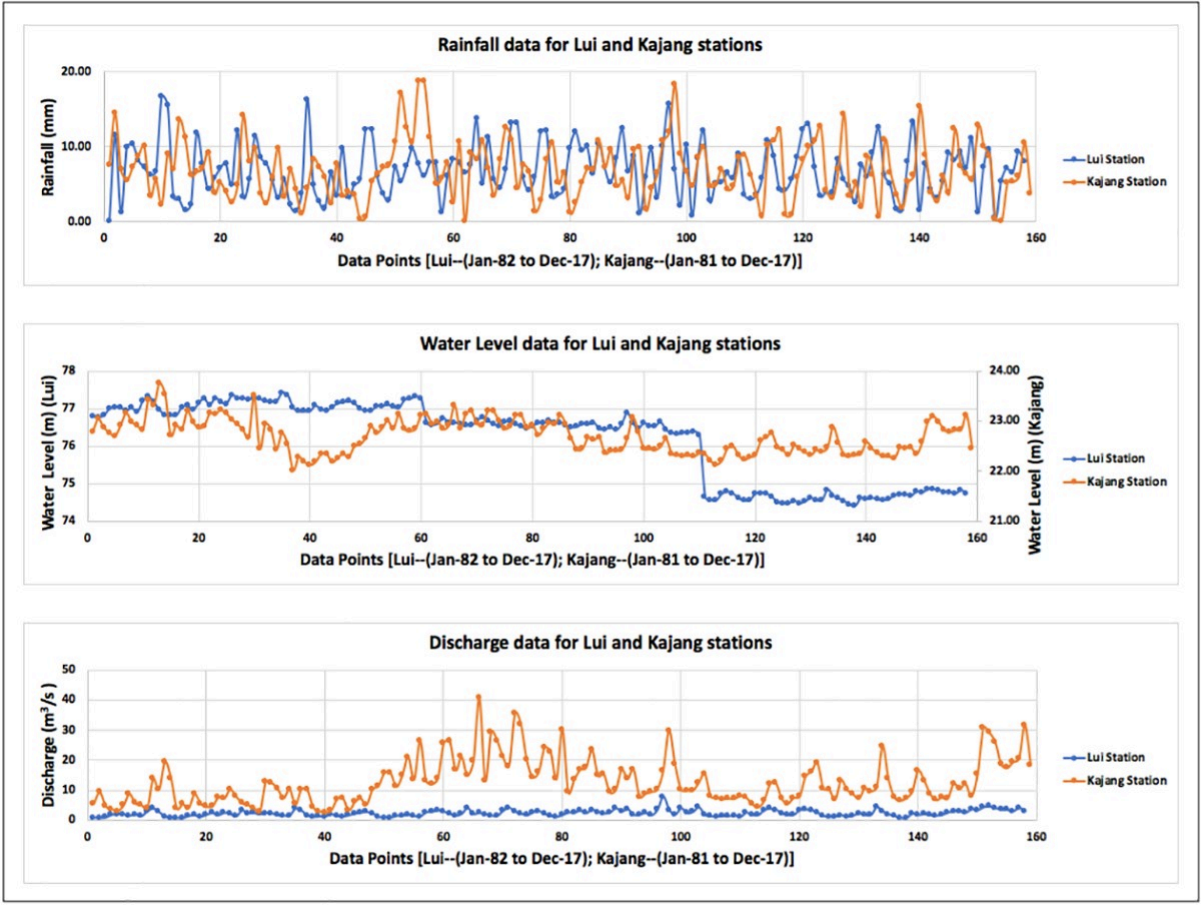
Plot of monthly average data of rainfall, water level and discharge.

Being an important step in data standardization [49], data received was pre-processed as it had some gaps with respect to time. There were also few irrelevant data such as, exceptionally high values. Such values were adjusted to the relevancy of the surrounding values. For interpolating the missing data, spline curve, normalized spline curve and ANN model were used. Spline curve and normalized spline curve did not provide satisfactory results, as these curves interpolated some negative values for nitrate-N and ammonia-N; which are not acceptable. Hence, feed-forward ANN model was used, which proved to be more accurate in interpolating the values. The interpolated monthly average data of nitrate-N and ammonia-N for stations Lui and Kajang are presented in Fig 3, with the data points arranged chronologically. Fig 4 represents the chronological data points of rainfall, water level and discharge for stations Lui and Kajang.

Statistical Analysis of the data (Table 1) reported that the average rainfall received at stations Lui and Kajang were approximately same (6.85 and 6.89 mm, respectively); with the maximum rainfall received at both the station as 16.70 and 18.75 mm, respectively. Water level and discharge differed at Lui and Kajang stations due to different geographical locations (mountainous and almost plane, respectively).

### Data division

For ANN multilayer modelling, input data has to be divided into three sets: training, validation and testing set [50]. The training set is used for adoption of the weights of neural network [51, 52], whereas the validation set is used for minimizing the overfitting of the network. ANN does not adjust its weights on the validation set. The testing set is used only for testing the final solution in order to confirm the actual predictive power of the network.

By default, ANN modeling system divides the input data as: 70% for training set, 15% for validation set and remaining 15% for testing set; by selecting randomly from the input set. Setting the division function as random, the network will randomly select different training, validation and testing set every time the network is trained. Hence, any conclusion cannot be drawn on the basis of accuracy by changing any internal parameter because training, validation and testing set keeps on changing every time the network is trained. Hence, for this study, the division function was selected as division index; in which the separate index numbers were provided for the three sets. These index numbers were selected from the input list such that all the three sets were statistically identical. These indices were selected randomly such that the mean values of all the three sets were close to each other. As suggested by Lagos-Avid and Bonilla [53] and Lu, Li [54], while selecting, it was ensured that the maximum and minimum output values were lying in the training set, so that network is trained for all patterns of the data available. After selecting the best set, it was stored and then used for all the network training for particular pollutant and station. Selection of indices was done separately and before training the neural network. Four set of data division were created which had the following percentage division:

1. Training = 75%, Validation = 12.5% and Testing = 12.5%
2. Training = 80%, Validation = 10% and Testing = 10%
3. Training = 85%, Validation = 7.5% and Testing = 7.5%
4. Training = 90%, Validation = 5% and Testing = 5%

### ANN training and parameter selection

GRNN, multilayer and RBFNN models were trained at different set of internal parameters. Separate training was carried out for nitrate-N and ammonia-N for stations Lui and Kajang. After training and testing the models on all combinations of the internal parameters, the optimum model was selected based on the regression values, mean square error and mean absolute error. Table 2 represents different values of internal parameters that were tested for ANN to get the most accurate model. Monthly average rainfall, water level and discharge were three inputs used in the model and also three different combinations of two inputs were used for training. Manually selected spread values were used for GRNN and RBFNN models. In multilayer, different models were developed having hidden layers 1, 2 and 3; having nodes in each hidden layer ranging from 2 to 10. Multilayer models were trained with epochs ranging from 100 to 1000. Training was done on Matlab platform; in which certain set of codes made it possible to train thousands of ANN models with each possible combination of different input variables and internal parameters.

**Table 2:** Different input and internal parameters for different ANN models.

**GRNN**	**Input:**	**Spread Values:**	**Data Division:**
	1. Three Input (RF, WL, Q) 2. Two Input (RF, WL) 3. Two Input (RF, Q) 4. Two Input (WL, Q)	0.001; 0.002; 0.004; 0.005; 0.006; 0.007; 0.008; 0.009; 0.01; 0.02; 0.04; 0.06; 0.08; 0.09; 0.1; 0.2; 0.4; 0.6; 0.8; 1; 2; 3; 4; 5	1. Training = 75% 2. Training = 80% 3. Training = 85% 4. Training = 90%
**Multilayer**	**Input:** 1. Three Input (RF, WL, Q) 2. Two Input (RF, WL) 3. Two Input (RF, Q) 4. Two Input (WL, Q)	**Hidden Layers:** 1, 2, 3 **Nodes:** 2, 3Δ.10 **Epochs:** 100, 200, 300. . .1000	**Data Division:** 1. Training = 75% 2. Training = 80% 3. Training = 85% 4. Training = 90%
**RBFNN**	**Input:** 1. Three Input (RF, WL, Q) 2. Two Input (RF, WL) 3. Two Input (RF, Q) 4. Two Input (WL, Q)	**Spread Values:** 0.01; 0.02; 0.04; 0.06; 0.08; 0.09; 0.1; 0.2; 0.4; 0.6; 0.8; 1; 2; 3; 4; 5	**Data Division:** 1. Training = 75% 2. Training = 80% 3. Training = 85% 4. Training = 90%

In comparison to the problems associated with the selection of the size of the input and output layers the issues associated with the size and number of the hidden layer are significantly more difficult to resolve. There are no strict guidelines available to select the correct number of hidden layers required or the needed number of hidden neurons as well. The exact requirements for each layer remain very application-specific despite the development of rule-o-thumb guidelines derived from the experience. This situation is in direct contrast to the process of defining the number of neurons in the input and output layer, where the stimulus and the desired response provide considerable guidance as to the number of input and output neurons required to perform a specified task.

The size of the hidden layer including the hidden neurons, more specifically the number of neurons (hidden) require a specified task that is intimately linked to the role of hidden neurons. In fact, the size of the hidden neurons affects not only how well the network is able to detect important features of the risk curves, but also its ability to generalize and make decisions based on curves which are not encountered during training. An indication of the importance of the architecture of the hidden layers is that hidden layers intermediately form the first response of the input data patterns. In case that there is an extra number of hidden neurons available within the layer, the final architecture might not be able to achieve generalization. On the other hand, a few numbers of neurons might lead to the inability to custom satisfactory and tolerate middle representations to be able to encode the final architecture to perceive and sense the important characteristics and attributes of the input pattern.

In the extreme, the loss of generalization due to too many hidden neurons can result in the grand-mothering effect. The grand-mothering effect refers to the condition where, if the number of hidden neurons is equal to the number of stimulus patterns employed during training, the network is capable in theory of perfectly memorizing these input patterns. However, in this situation, the network does not learn to detect patterns in the stimulus, but rather uses each neuron in the hidden layer to memorize the desired response of one of the training stimuli. Without the ability to detect important features of a stimulus, the network is unable to generalize.

Currently, the most common approach available to identify the appropriate number of hidden neurons in the hidden layer is the trial-and-error approach. Using the trial-and-error approach is mainly to try a training process with a different number of neurons in the hidden layer and evaluate the model’s outputs compared with the desired actual outputs since the feature of the input data and the aptitude to generalize these results. The optimal architecture of the network is the network that could achieve good results and sense the important characteristics of the input pattern with a minimal number of hidden neurons.

While the experimental approach to find the optimal number of hidden neurons can be implemented successfully, it is very time consuming and requires the investigation of a large number of neural networks. An alternative procedure for finding the optimal number of neurons could be adjusted. This procedure, referred to as the dynamic-node-creation method, progressively adds neuron to the hidden layer whenever the network can no longer be improved using the current number of hidden neurons. A practical metric to determine how close the network's output is to the desired response is the sum of the squared differences (D_t_). This progressive addition to neurons is accomplished by adding a new neuron when any improvement to the training metric *D_t_*, is insignificant. Letting *D_t_* denotes the value of the training metrics at iteration t, the following equation shows the process for adding new neuron:

$$\frac{\left\lfloor D_{t}-D_{t-\varepsilon }\right\rfloor }{D_{t_{o}}}<\Delta T,\;(t\geq t_{o}+\varepsilon )$$ (1)

Where t_o_ is iteration index at the prior neurons number, ε represents the number of iterations through the error curve searching slope *D_t_* could be computed, and Δ*T* denotes the slope of the trigger. The optimal final condition as presented in Eq (1) guarantees that at best training iterations ε have been carried out before any further new additional neuron is appended. The stopping criteria for this procedure are achieved when *D_t_* is adequately small or the performance goal of convergence is attained.

The convergence of the neural network (when the number of neurons in the hidden layer is at its optimum) is best assessed using the maximum squared difference (errors) at any time t. Mathematically, the largest squared error is:

$$D_{max}=\underset{\mu ,i}{\max}\left[{\left(S_{L_{i}}^{\mu }-\zeta_{i}^{\mu }\right)}^{2}\right],$$ (2)

When the largest squared error experiences a drastic drop, the optimal number of neurons has been identified. The objective of the training session is to obtain an output response $S_{L_{i}}^{\mu }$, i = 1,Δ, NL, that is ideally the same as the desired response $\zeta_{i}^{\mu }$, i = 1,Δ, NL, where NL is the number of neurons required to define the response.

### Performance criteria

For a neural network, to produce accurate result, the selection of hidden layers and its neurons and number of inputs are essential. Analysis was based on the regression values (Eq 3) of training, validation and testing. Accuracy of the model cannot be decided based on the regression values alone [55]. The regression values give the statistical measure of the data fitting to the best fit line but cannot indicate the deviation of the predicted data from the observed data. Hence, mean absolute error (MAE) (Eq 4), mean square error (MSE) (Eq 5), plot of the observed and the predicted values, plot of relative error percentage values (Eq 6) and plot of models on Taylor diagram were also considered in the process of optimum model selection. Taylor diagrams were drawn on the basis of the testing standard deviation, testing mean square error and testing correlation. In Taylor diagram, the model that is close to the actual point is the optimum model. The actual point is the observed value of the pollutants (nitrate-N or ammonia-N), which has a definite standard deviation, a correlation value of 1 and a mean square error of zero. The closest model to the actual point has the standard deviation near to the observed values and correlation, with the observed values, close to 1 and least mean square error; making the model best fit for predicting the actual values. Equations for the performance criteria are given hereafter:

- Regression Values:

$$r=\frac{n(\sum xy)-(\sum x)(\sum y)}{\sqrt{\left[n\sum x^{2}-{\left(\sum x\right)}^{2}][n\sum y^{2}-{\left(\sum y\right)}^{2}\right]}}$$ (3)

- Mean Absolute Error:

$$MAE=\frac{1}{n}\sum_{i=1}^{n}\left|x-y\right|$$ (4)

- Mean Square Error:

$$MSE=\frac{1}{n}\sum_{i=1}^{n}{\left(x-y\right)}^{2}$$ (5)

- Relative Error Percentage:

$$RE=\frac{\left|x-y\right|}{x}*100$$ (6)

Where, in this study, n = number of data points, x = observed data points, and y = predicted data points

## Results

**Fig 5:**
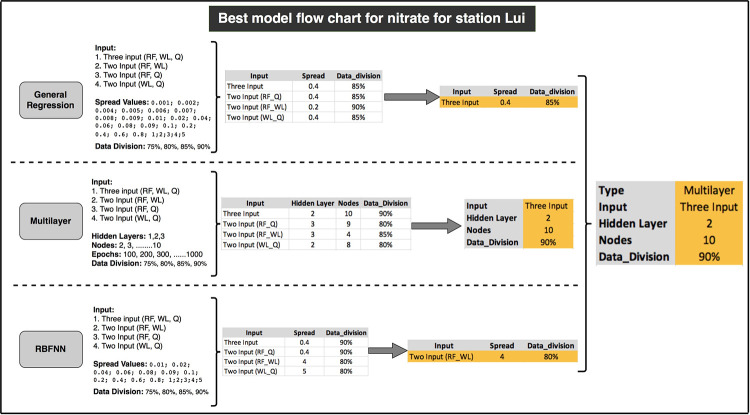
Flow chart for the model selection for nitrate-N at Lui station.

Training of GRNN, multilayer and RBFNN models with different set of parameters and input variables resulted in tens of thousands of networks, each with different combinations of parameters and different results. These models were analyzed based on the performance criteria, sequentially, to bring out the optimum model. Initially, the regression values were used to filter out thousands of low regression valued model, followed by examining high regression valued models on other analysis parameters to sort out the optimum one. The main aim of the analysis was to bring out four optimum neural network models for nitrate-N and ammonia-N each for the stations Lui and Kajang. Fig 5 represents the flow chart for the selection procedure of the optimum model for nitrate-N at Lui station. Same procedure was followed for the selection of optimum ANN model for ammonia-N at Lui station, and nitrate-N and ammonia-N at Kajang station.

**Fig 6:**
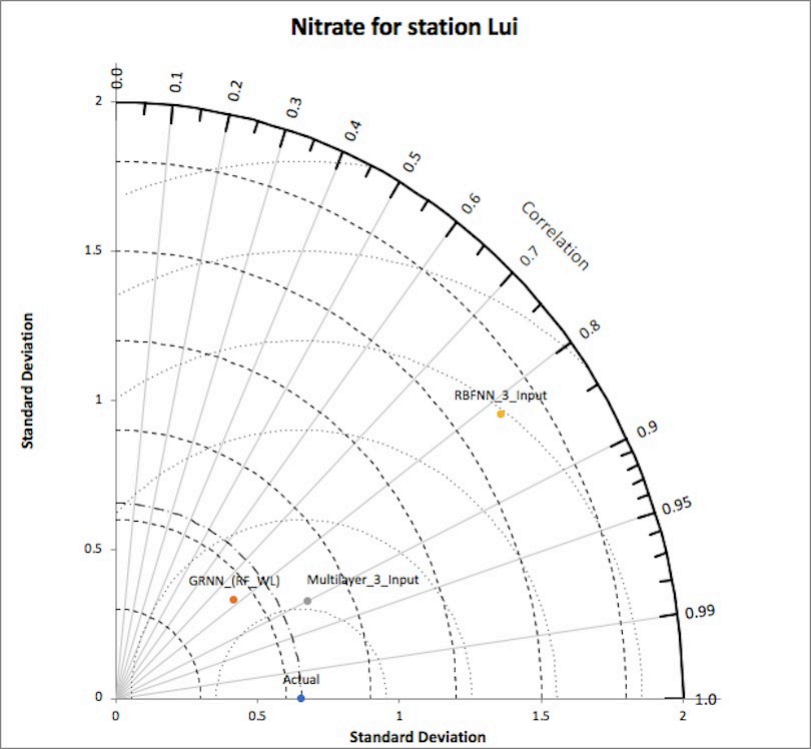
Taylor diagram for nitrate-N (Lui).

**Fig 7:**
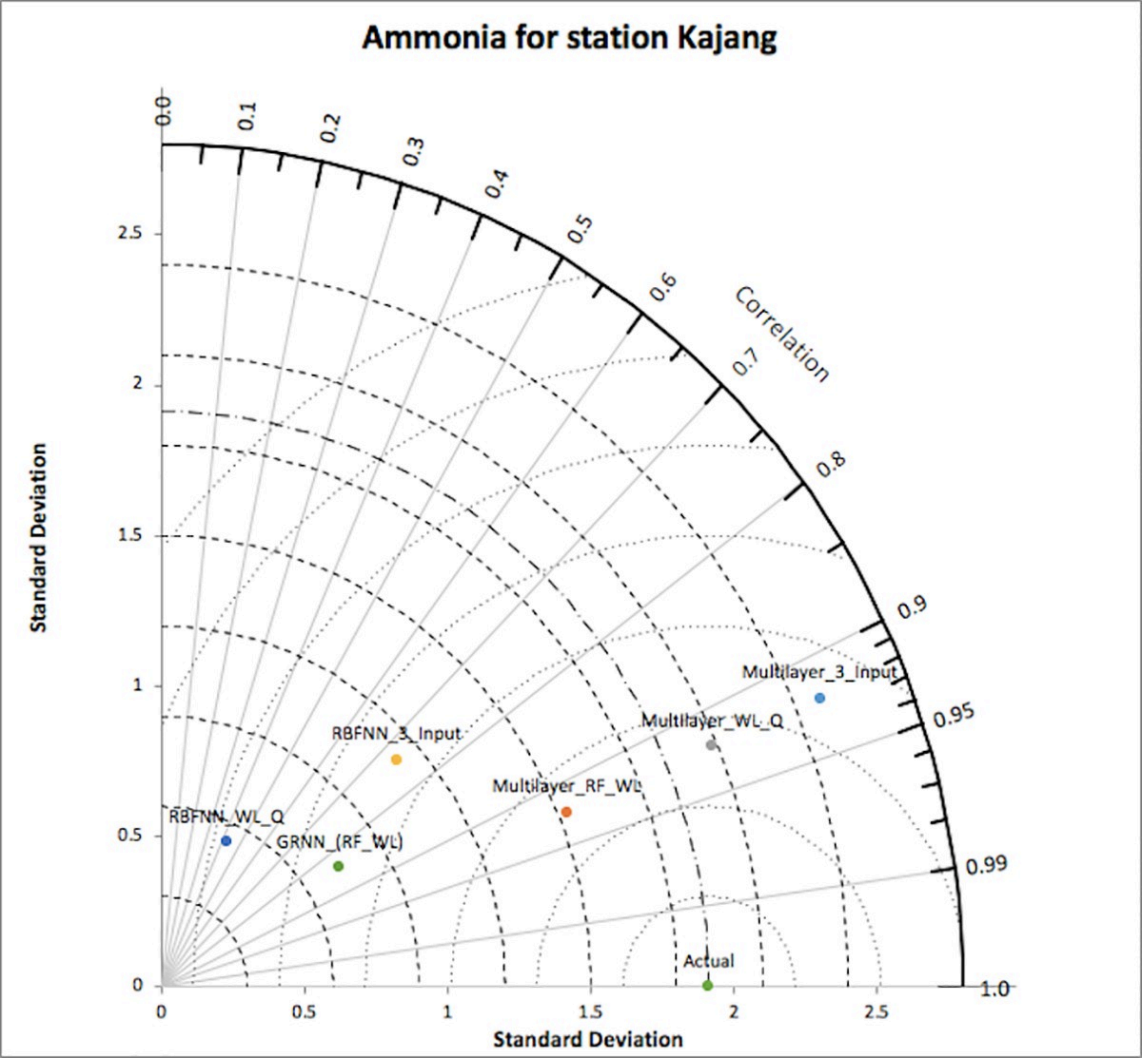
Taylor diagram for ammonia-N (Kajang).

Fig 6 represents the Taylor diagram of models for nitrate-N for at Lui station; which clearly displays that the multilayer model with three input and general regression model with input as RF and WL, are close to the actual point but the relative error percentage plot, and the plot of observed vs predicted values for multilayer model were acceptable over general regression model. Hence, the multilayer model with three inputs is considered to be the optimum in comparison with other models. Fig 7 represents the Taylor diagram of models for ammonia-N at Kajang station. It represents that multilayer models with three inputs, with input as RF and WL and with input as WL and Q are close to the actual point. On analyzing the relative error percentage plots and plot of observed vs predicted values, it was found that the multilayer model with three inputs has the promising results over other models. Hence, this model was considered as the optimum in comparison with others. Similar procedures were followed for the other two models i.e., for ammonia-N for station Lui and for nitrate-N for station Kajang.

It is evident that there cannot be one universal model which predicts the desired hydrological parameters for different geographical locations. Model trained on the data of one particular location cannot predict the desired variable of other locations, as all locations differ hydrologically, and historical data have different patterns which the model trained at different location may have not seen. Hence, four different models have been selected, two for each location corresponding to nitrate-N and ammonia-N. Table 3 represents the configuration and regression values of final selected models for Lui and Kajang stations for nitrate-N and ammonia-N. All the selected models are multilayer ANN with overall regression value more than 0.90 and input data division as 90% for training, 5% for validation and 5% for testing. Nash-Sutcliffe Efficiency for all the four optimum models are close to 1; which indicates that models have efficiently predicted the actual values.

**Table 3:** Optimum ANN models for Lui and Kajang stations.

Station		Lui		Kajang	
Nitrogen Compound		Nitrate-N	Ammonia-N	Nitrate-N	Ammonia-N
**Parameters**	**Type**	Multilayer	Multilayer	Multilayer	Multilayer
	**Inputs**	Three Input (RF, WL, Q)	Three Input (RF, WL, Q)	Three Input (RF, WL, Q)	Three Input (RF, WL, Q)
	**Hidden Layer**	2	3	3	2
	**No. of Nodes**	10	7	8	9
	**Epochs**	300	200	1000	1000
	**Training data**	90%	90%	90%	90%
**Accuracy**	**Overall Regression**	0.98	0.968	0.92	0.98
	**Training Regression**	0.99	0.988	0.99	0.99
	**Validation Regression**	0.95	0.704	0.92	0.95
	**Testing Regression**	0.90	0.65	0.61	0.92
	**Mean Absolute Error**	0.0978	0.017	0.771	0.173
	**Mean Square Error**	0.027	0.0013	7.09	0.121
	**Nash-Sutcliffe Efficiency**	0.9666	0.9457	0.9588	0.9715

**Fig 8:**
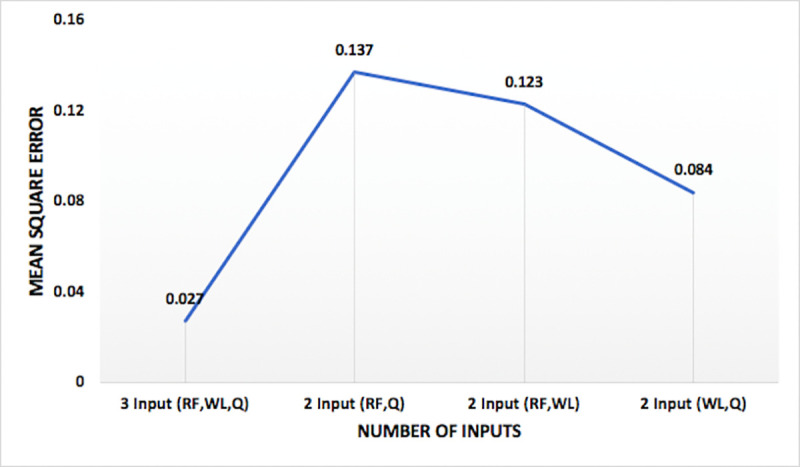
Comparison of mean square error for different input vectors for nitrate-N at station Lui.

**Fig 9:**
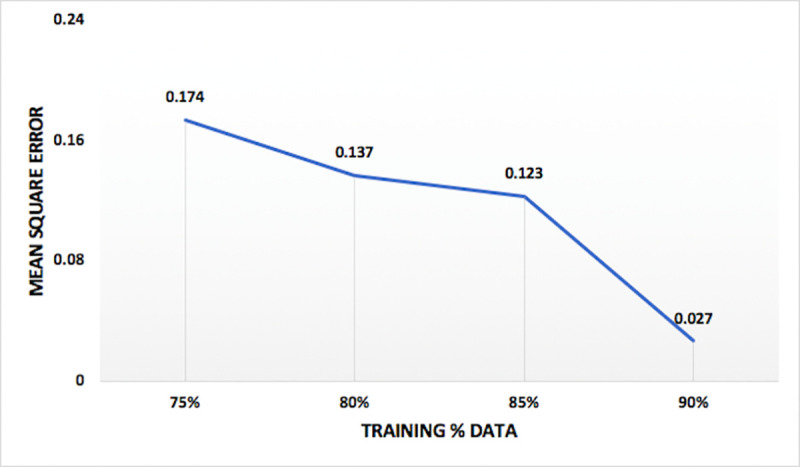
Comparison of mean square error for different training data division percentage for nitrate-N at station Lui.

**Fig 10:**
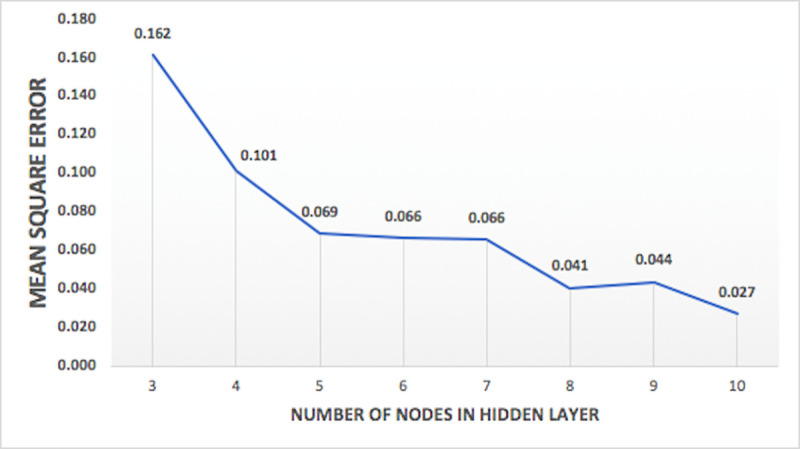
Plot of variation of mean square error against number of nodes in hidden layers for nitrate-N at station Lui.

**Fig 11:**
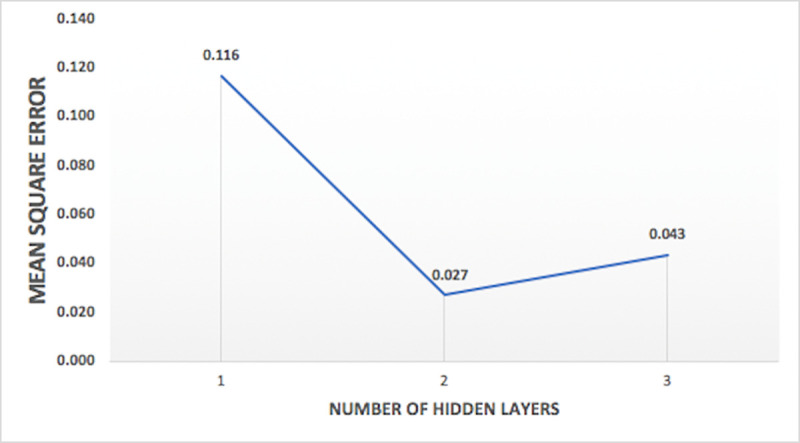
Plot of variation of mean square error against number hidden layers for nitrate-N at station Lui.

Models were tested for different combination of input vectors and internal parameters, as given in Table 2. Model performance, measured with mean square error, varied with variations in different internal parameter and input vectors. Analyzing the model performance by varying number of inputs, it is observed that model has least mean square error when all the three input vectors are used. Hence, three inputs (RF, WL, Q) are selected for optimum models. One of the comparisons between the four set of input vectors on the basis of mean square error of the model for nitrate-N at station Lui, is shown in Fig 8. Variation of performance of the model on the basis of percentage data division seems to follow a pattern of training a model with more percentage of data will lead to better results. Hence, the model with 90% training data has least mean square error and is used for optimum models. The comparison between the percentage data divisions on the basis of mean square error of the model for nitrate-N at the station Lui, is shown in Fig 9. Variation of performance of the models on the basis of number of nodes in hidden layers is presented in Fig 10 and the variation of the performance of the models on the basis of number of hidden layers is shown in Fig 11. The concept of increasing the number of hidden layers and number of nodes in the model, as explained earlier, is to increase the complexity of the network which helps the model to learn different patterns in the target data. Beyond a certain number of hidden layer and nodes in it, network becomes over complexed leading to the decrease in the performance of the model. Within the selected range of number of nodes, for this study, it is observed that the mean square error is decreasing with increase in the nodes. And for the hidden layers, the minimum mean square error is obtained at two hidden layers, beyond which network seems to have become over complexed as the mean square error increased for three hidden layers.

**Fig 12:**
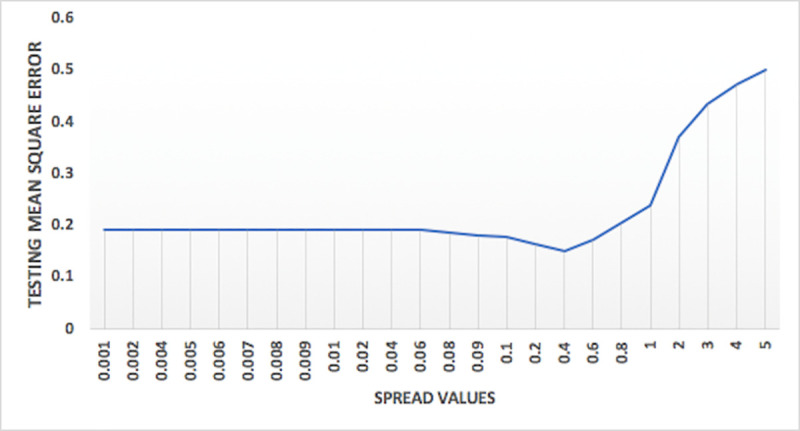
Plot of variation of testing mean square error against spread values for general regression model for nitrate-N at station Lui.

**Fig 13:**
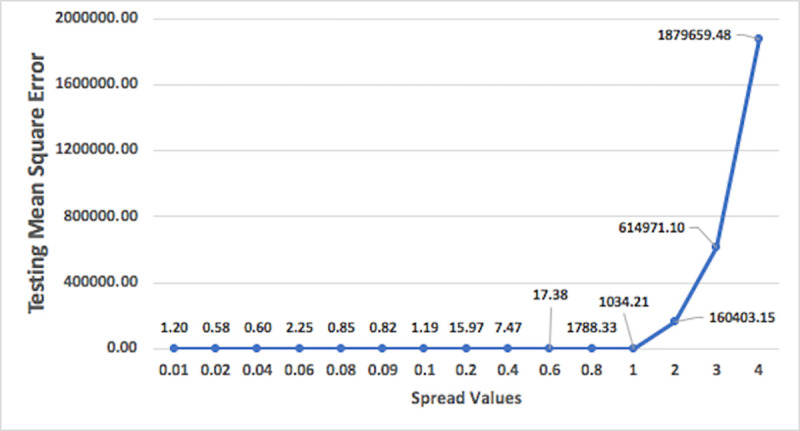
Plot of variation of testing mean square error against spread values for RBFNN model for nitrate-N at station Lui.

**Fig 14:**
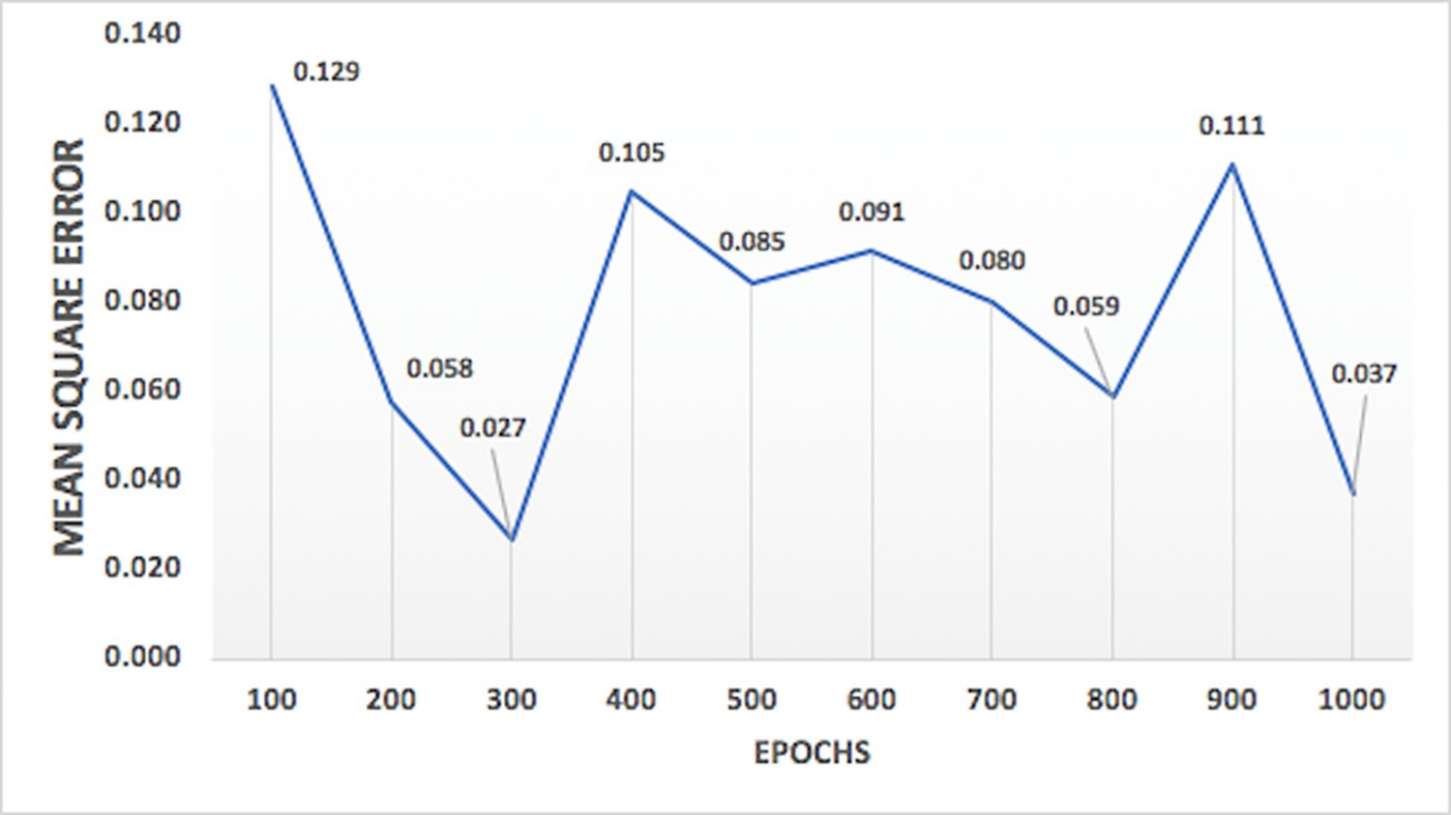
Plot of variation of mean square error against epochs for nitrate-N at station Lui.

Variation of performance of models on the basis of spread values for general regression and RBFNN models are shown in Figs 12 and 13, respectively. As shown in the Figs 12 and 13, the testing mean square error for these models are decreasing with increase in the spread values and after a certain point it increases with further increase in spread values, leading to the identification of a spread value having better accuracy and suitable for optimum model. Fig 14 shows the plot of the variation of mean square error against the number of epochs. The concept of changing training epochs is to allow the model to train sufficient number of iterations and also to stop before the model begins overtraining. For the model predicting nitrate-N at station Lui, the optimum epochs obtained from Fig 14 is 300, as the model delivers least mean square error indicating that model is trained with sufficient number of iterations without being over-trained. The number of epochs beyond which model starts overtraining depends on the complexity of the network.

## Discussion

While filtering out thousands of models, it was observed that some models of GRNN and RBFNN performed well with training regression of more than 0.98 but did not perform satisfactorily in testing when new input data was fed into the model, which the model was not exposed to in the training process. This led to low regression values for testing and high mean square error values. In the selection process, the main focus was on the testing results of the model, which represents the exact ability of the model to predict the actual values. The possible explanation of the low testing regression and high mean square error of those GRNN and RBFNN models is overfitting, which generally lead to high training regression values and low testing regression values.

As shown in Table 3, the testing regression values for ammonia-N for the Lui station and for nitrate-N for the Kajang station were 0.65 and 0.61, respectively, which are considerably low in comparison with testing regression values for other models. The reason for the low testing regression values lies in the correlation of the input variables mainly with the output variables. The data obtained for the study showed good correlation for nitrate-N for the Lui station and satisfactory correlation for ammonia-N for the Kajang station but low values for the nitrate-N for the Kajang station and for ammonia-N for the Lui station. The correlation for the Lui station for RF, WL and Q with ammonia-N were 0.57, 0.61 and 0.61 respectively and for the Kajang station for RF, WL and Q with nitrate-N were 0.69, 0.75 and 0.67 respectively. Corresponding to the low values of the correlation and other unaccountable natural parameters, upon which concentration of these compound depends, the model failed to establish the relation between the input variables and the output variables, leading to low testing regression.

**Fig 15:**
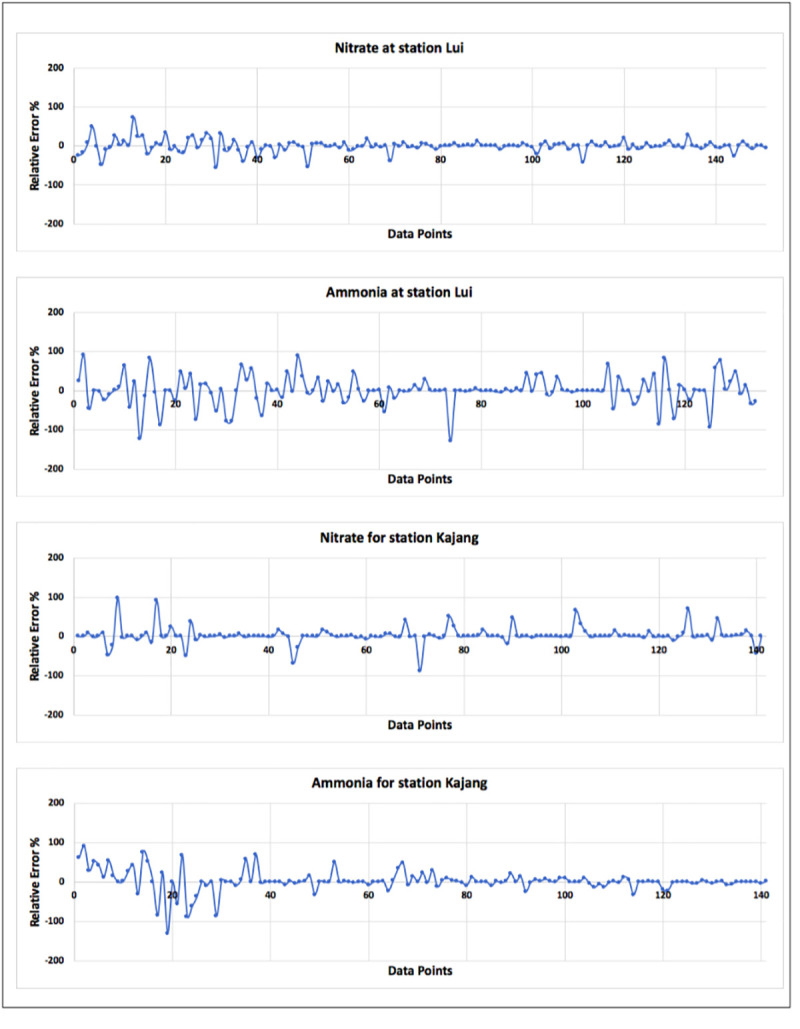
Relative error percentage plot for optimum selected model.

Fig 15 represents the percentage relative error of the four optimum models selected for stations Lui and Kajang for nitrate-N and ammonia-N. Data points in these figures are arranged chronologically. Relative error figures represent that the model generated more error for the data recorded in earlier days i.e. in 1980s. Some of these errors reached near 100%, but the maximum number of errors were close to zero-percentage line. High error values could be brought close to the zero-percentage line using deep learning methods.

**Fig 16:**
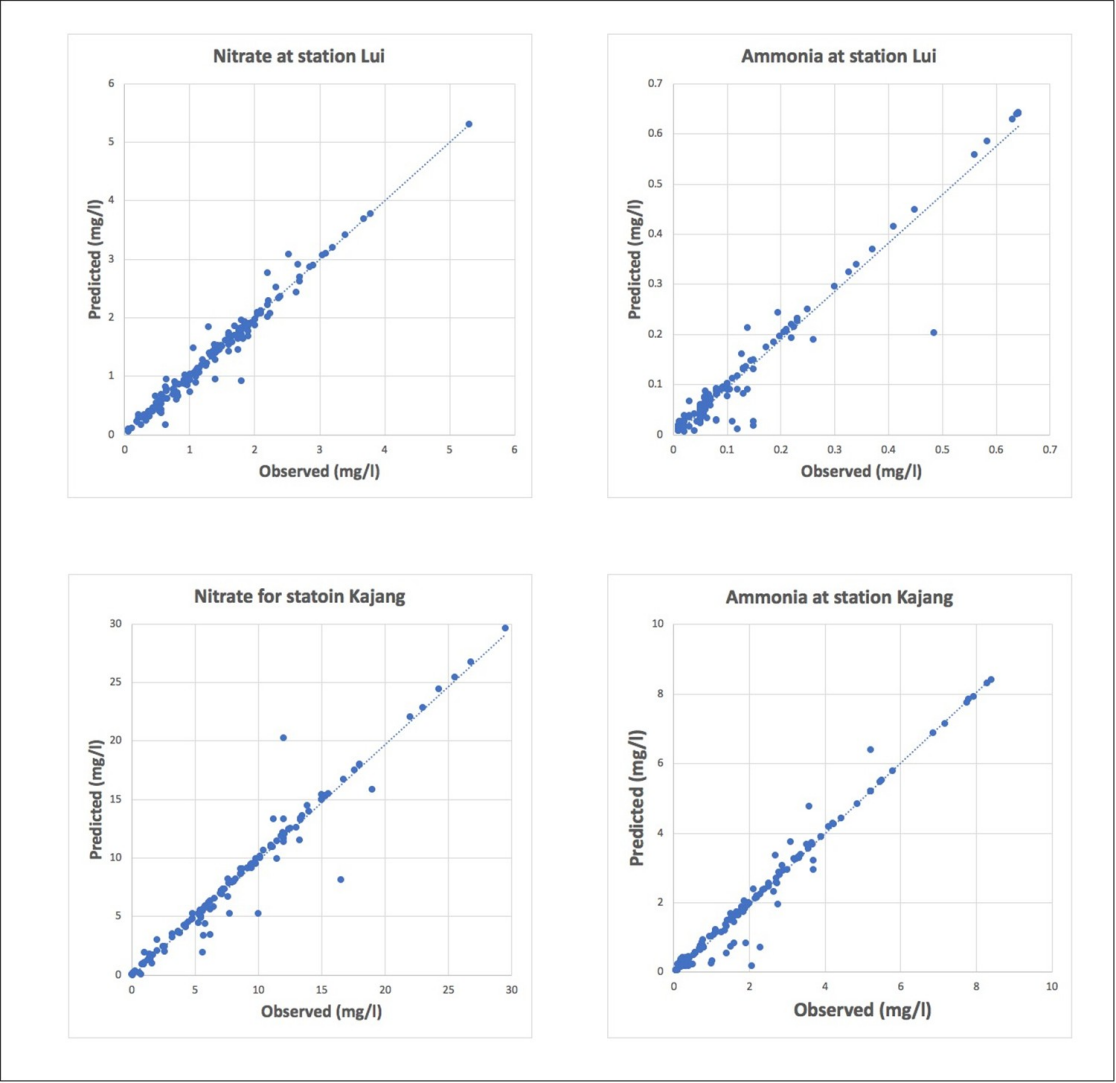
Observed versus predicted plot for optimum selected models.

Fig 16 represents the plot of the observed vs predicted values for the optimum selected models. The trend line formed approximately 45° for all the selected models and also, nearly all the points lied near to the trend line. This indicated that the predicted values were very close to the observed values. Hence, making these models optimum for predicting the monthly average nitrate-N and monthly average ammonia-N for the Langat River.

According to Chitsazan, Nadiri [39], the sources of uncertainty in model prediction lies in the uncertainty in model inputs, model structure, weights and biases. However, most important source is the uncertainty in the model inputs. In the current study, model inputs had few time gaps. Some of those minor time gaps were covered with interpolated values, thus introducing some amount of uncertainty in model inputs. Average uncertainty in the prediction can be calculated using the following equation [56]:

$$\sigma =\frac{1}{n}\sum_{i=1}^{n}\left(\frac{\left|x-y\right|}{x}\right)*100$$ (7)

where: σ = average uncertainty percentage, n = number of data points, x = observed data points, and y = predicted data points

Uncertainty increases at every level of calculation or prediction performed using the data already having some amount of uncertainty. Interpolation of the data, used in this study, for obtaining the missing values had introduced some amount of uncertainty in the input data, which may have multiplied in the output values after prediction. To reduce the amount uncertainty in the output values it is advised to try to minimize it from the initial stage of processing the raw data obtained for the study.

**Fig 17:**
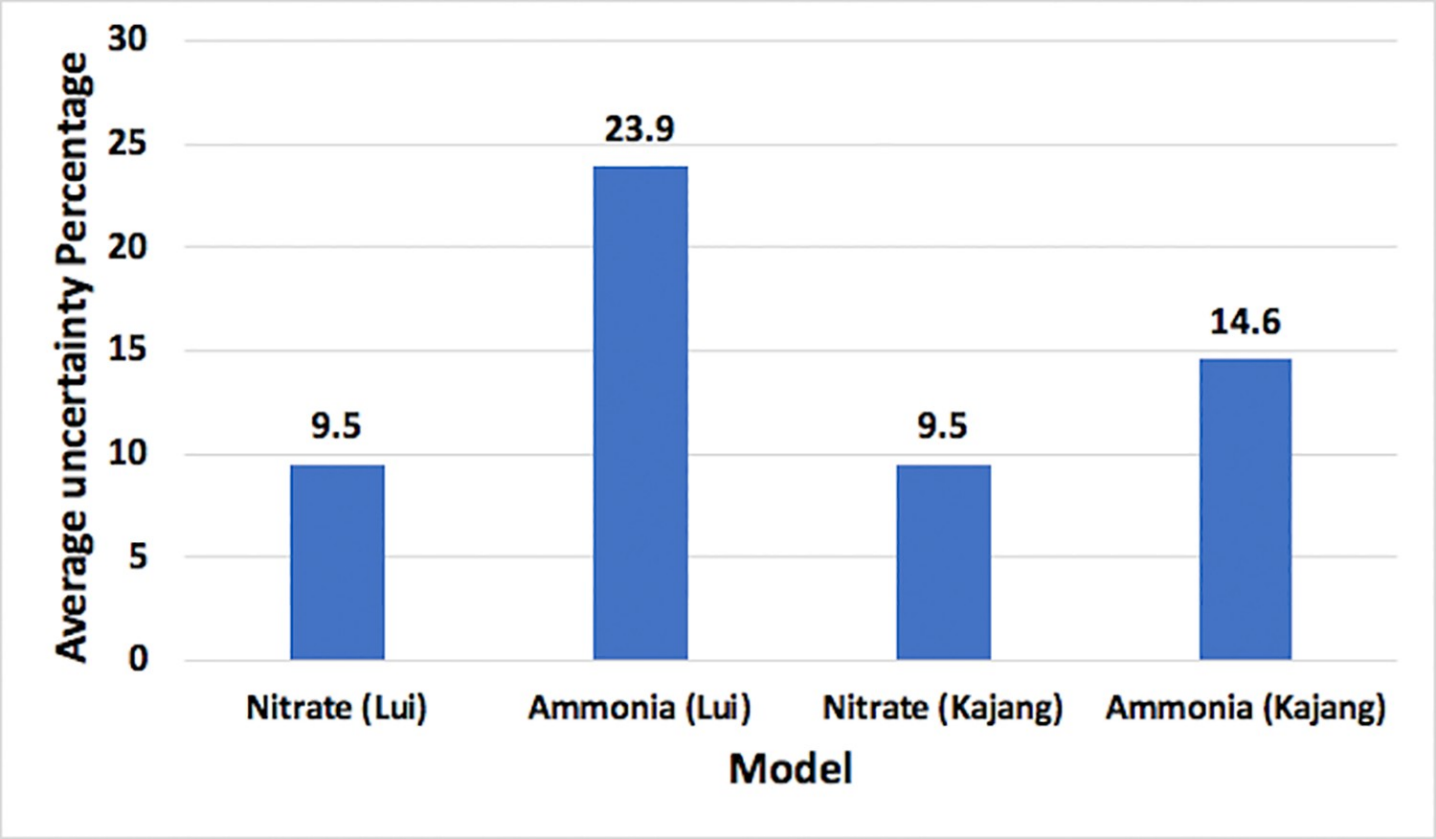
Average uncertainty of different models.

Average uncertainty of all the four selected optimum models, calculated by Eq (7), are shown in Fig 17. Model predicting nitrate-N for both the stations, Lui and Kajang, show less uncertainty of 9.5%. Ammonia-N model at station Lui shows highest uncertainty of 23.9%. These models seem appropriate for nitrate-N and ammonia-N prediction at station Lui and Kajang.

Selected models provide improved results when compared with the existing models available in literature. Analyzing the accuracy of the nitrate-N-predicting models (Table 4), existing in literature, it can be observed that current study models provide results with better regression values. Anctil, Filion [24] used stacked multilayer perceptron to model nitrate-nitrogen flux in streams and had the efficiency index of 0.888. Suen and Eheart [15] implemented back-propagation and radial basis function neural network for predicting nitrate-N concentration in streams. Sharma, Negi [23] predicted nitrate-N concentration in drainage water. Markus, Hejazi [25] predicted weekly nitrate nitrogen, in streams, using evolutionary polynomial regression, Naïve Bayes model and back-propagation neural network.

**Table 4:** Comparison of current study results with literature.

	Current Study				Literature			
**Station/ Author**	Lui	Lui	Kajang	Kajang	Anctil et al. (2009)	Suen and Eheart (2003)	Sharma et al. (2003)	Markus et al. (2010)
**Prediction Variable**	Monthly Average Nitrate-N	Monthly Average Ammonia-N	Monthly Average Nitrate-N	Monthly Average Ammonia-N	Nitrate- nitrogen flux	Nitrate concentration	Nitrate concentration	Weekly nitrate- nitrogen
**Accuracy**	**Regression:** • **Overall:** 0.98 • **Training:** 0.99 • **Validation:** 0.95 • **Testing:** 0.90 **MAE:** 0.0978**MSE:** 0.027**Nash-Sutcliffe Efficiency**: 0.9666	**Regression:** • **Overall:** 0.968 • **Training:** 0.988 • **Validation:** 0.704 • **Testing:** 0.65 **MAE:** 0.017**MSE:** 0.0013**Nash-Sutcliffe Efficiency:** 0.9457	**Regression:** • **Overall:** 0.92 • **Training:** 0.99 • **Validation:** 0.92 • **Testing:** 0.61 **MAE:** 0.771**MSE:** 7.09**Nash-Sutcliffe Efficiency:** 0.9588	**Regression:** • **Overall:** 0.98 • **Training:** 0.99 • **Validation:** 0.95 • **Testing:** 0.92 **MAE:** 0.173**MSE:** 0.121**Nash-Sutcliffe Efficiency:** 0.9715	**Efficiency index** = 0.888	Overall accuracy: • **Method one:** • BPNN = 0.784 • RBFNN = 0.752 • **Method two:** • BPNN = 0.832 • RBFNN = 0.832 • **Boolean output (Method two)**• BPNN = 0.866 • RBFNN **=** 0.893	**Correlation coefficient** • **RBFNN** • Tillage = 0.8079 • No tillage = 0.6911 • **BPNN** • Tillage = 0.8017 • No Tillage = 0.6635	**RMSE for ANN:** • Training = 0.787 mg/l • Testing = 0.935 mg/l **RMSE for evolutionary polynomial regression (EPR):** • Training = 0.991 mg/l • Testing = 1.010 mg/l **Critical success index for NBM:** • Training = 0.286 • Testing = 0.188

## Conclusion

Selection of the appropriate internal parameters for the ANN models along with the relevant input variables are essential to ensure accuracy. This paper discussed the selection procedure of those internal parameters and input variables for the ANN model for predicting the monthly average nitrate-N and monthly average ammonia-N levels in the Langat River in Selangor, Malaysia. Also, the discussion about the variation of performance response of the model against the variation of different internal parameters and input variables is also included. Among the three model architectures (i.e. GRNN, multilayer and RBFNN), the multilayer model performed very well for nitrogen and ammonia-N prediction. Among the various sets of internal parameters and inputs, selected models have three input variables (RF, WL, and Q) and the data division for training as 90%, validation as 5% and testing as the remaining 5%. The minimum overall regression of the four selected optimum models is 0.92. Nash-Sutcliffe Efficiency for the selected optimum models are very close to 1. Maximum relative error percentage points are close to zero-percentage line, with few data point approaching more than 100%; which can be brought back to the zero-percentage line by using deep leaning method. Based on the results and their comparison between different sets of training data divisions, it can be stated that higher percentage of data for training will eventually lead to better accuracy of the model.
